# Epidemiology of Enterovirus D68 in Ontario

**DOI:** 10.1371/journal.pone.0142841

**Published:** 2015-11-23

**Authors:** Adriana Peci, Anne-Luise Winter, Bryna Warshawsky, Tim F. Booth, AliReza Eshaghi, Aimin Li, Stephen Perusini, Romy Olsha, Alex Marchand-Austin, Erik Kristjanson, Jonathan B. Gubbay

**Affiliations:** 1 Public Health Ontario Laboratory, 661 University Ave, Toronto, Ontario, Canada; 2 Public Health Ontario, 480 University Ave, Toronto, Ontario,Canada; 3 National Microbiology Laboratory, Public Health Agency of Canada, 1015 Arlington St., Suite H5300, Winnipeg, Manitoba, Canada; Naval Research Laboratory, UNITED STATES

## Abstract

In August 2014, children’s hospitals in Kansas City, Missouri and Chicago, Illinois notified the Centers for Disease Control and Prevention (CDC) about increased numbers of pediatric patients hospitalized with severe respiratory illness (SRI). In response to CDC reports, Public Health Ontario Laboratories (PHOL) launched an investigation of patients being tested for enterovirus D-68 (EV-D68) in Ontario, Canada. The purpose of this investigation was to enhance our understanding of EV-D68 epidemiology and clinical features. Data for this study included specimens submitted for EV-D68 testing at PHOL from September 1, 2014 to October 31, 2014. Comparisons were made between patients who tested positive for the virus (cases) and those testing negative (controls). EV-D68 was identified in 153/907 (16.8%) of patients tested. In the logistic regression model adjusting for age, sex, setting and time to specimen collection, individuals younger than 20 years of age were more likely to be diagnosed with EV-D68 compared to those 20 and over, with peak positivity at ages 5–9 years. Cases were not more likely to be hospitalized than controls. Cases were more likely to be identified in September than October (OR 8.07; 95% CI 5.15 to 12.64). Routine viral culture and multiplex PCR were inadequate methods to identify EV-D68 due to poor sensitivity and inability to differentiate EV-D68 from other enterovirus serotypes or rhinovirus. Testing for EV-D68 in Ontario from July to December, 2014 detected the presence of EV-D68 virus among young children during September-October, 2014, with most cases detected in September. There was no difference in hospitalization status between cases and controls. In order to better understand the epidemiology of this virus, surveillance for EV-D68 should include testing of symptomatic individuals from all treatment settings and patient age groups, with collection and analysis of comprehensive clinical and epidemiological data.

## Introduction

In August 2014, children’s hospitals in Kansas City, Missouri and Chicago, Illinois notified the Centers for Disease Control and Prevention (CDC) about increased numbers of pediatric patients hospitalized with severe respiratory illness (SRI)[1]. Laboratory testing identified enterovirus D68 (EV-D68) in respiratory specimens from most of these children. From mid-August 2014 to January 15, 2015, 1,153 EV-D68 cases in 49 US states were confirmed by CDC. Reports indicated many of these children had a history of asthma or wheezing [2]. A total of 214 EV-D68 positive patients were confirmed in Canada from August to October, 2014. As of December 9, 2014, cases were reported from Alberta, British Columbia, Manitoba, New Brunswick, Nova Scotia, Ontario, Quebec, Prince Edward Island and Saskatchewan [3].

EV-D68 is a member of the *Picornaviridae* family [1]. Although EV-D68 is an enterovirus, it shares biological characteristics with rhinoviruses such as improved growth at low temperatures and instability at low pH [4, 5] 5. In addition, similar to rhinoviruses, EV-D68 has been isolated primarily from respiratory specimens rather than from stool specimens of patients with aseptic meningitis, which is characteristic of most other enteroviruses [4, 5].

EV-D68 has primarily been associated with respiratory disease since its discovery in 1962 [4, 5]. In the literature, the spectrum of illness attributed to EV-D68 ranges from asymptomatic to severe respiratory infection requiring hospitalization particularly in children with previous history of asthma or wheezing; however the full spectrum of disease is not yet known [4, 6–8]. A possible association with neurological syndromes such as acute flaccid paralysis was noted during the 2014 increase in the US of EV-D68, and some individuals presenting with acute flaccid paralysis (now classified as acute flaccid myelitis) had EV-D68 detected in respiratory specimens. A recent investigation of hospital patients presenting with acute flaccid myelitis [9] reported an association of EV-D68 infection and acute flaccid myelitis; however, the connection between EV-D68 infection and neurological syndromes remains unclear and causality has not been determined [10,11].

It is difficult to know the true burden of disease related to EV-D68 since EV infections are not reportable in North America and some patients infected with the virus don’t seek medical care or even when they do, routine testing does not differentiate this serotype [12]. Thus, the reported incidence of infection is likely an underestimation of the true incidence [1].

While more recent investigations reported on EV-D68 infection in children who present to hospital [13], the virus has also been identified in both children and adults seeking community-based care [9,14]. Consistent with previous reports [15], a higher proportion of individuals in whom the virus was identified were male.

In response to more recent reports from the US, Public Health Ontario Laboratories (PHOL), in Ontario, Canada launched an investigation to document clinical and laboratory data on patients being tested for EV-D68. The purpose of this investigation was to enhance our understanding of EV-D68 epidemiology and associated clinical features.

## Materials and Methods

Specimens are collected from patients by submitters and sent to PHOL for testing as part of routine clinical service. These data are also used for routine laboratory surveillance, which is a mandate of Public Health Ontario. Therefore, consultation with our organization’s privacy office or ethics committee was not required. To protect patient privacy and confidentiality, data are reported in an aggregated anonymized format.

Data for this study included specimens submitted for EV-D68 testing at PHOL from patients who presented with respiratory symptoms in different health care settings across Ontario, from September 1, 2014 to October 31, 2014. For the purpose of this investigation, cases were individuals who tested positive for the virus whereas controls were individuals who tested negative for EV-D68; the term “patients” refers to both cases and controls.

PHOL has had the capacity to test respiratory specimens for EV-D68 since September 24, 2014. Prior to that time, all EV-D68 testing on behalf of PHOL was performed at the National Microbiology Laboratory, (NML) Canada’s reference laboratory. At both laboratories, primary specimens underwent total nucleic acid extraction using the bioMérieux NucliSENS^®^ easyMAG^®^ protocol (bioMérieux Inc. Marcy I’Etoile, Rhone, France). Screening of clinical specimens for EV-D68 at PHOL was performed using a real time PCR assay targeting the 5’NTR region)[16]. Subsequently, all positive specimens were subjected to a semi-nested reverse transcriptase polymerase chain reaction (RT-PCR) targeting the VP1 gene coding for the capsid protein followed by Sanger sequencing with Bigdye V3.1 kit (Life Technologies Inc. California, USA) to amplify of a 375 bp partial VP1 region for serotype identification)[17]. A similar testing method was performed at NML, with sequencing performed by the Genomics core facility at NML [18]. Sequences were assembled using Sequencer 5.2.4 and compared to VP1 sequences in GenBank using BLAST. NML also employed an EV-D68 strain-specific real-time reverse transcription PCR test [19]. Testing at PHOL continued until December 1, 2014 and it was also performed retrospectively for some patients from whom specimens were submitted before September.

A selection of specimens included in this investigation were also tested by viral culture and/or multiplex PCR as per PHOL’s routine laboratory testing algorithm. For virus culture testing, rhesus monkey and WI-38 (and /or MRC5) cell lines were used to isolate viruses from different specimens including respiratory specimens. Growth of viruses (cytopathogenic effects) were confirmed using fluorescent monoclonal antibodies (MAbs). Multiplex PCR (Seeplex1 RV, Seeplex1 RV15 ACE; Seegene, USA) were used to test for adenovirus, coronavirus, enterovirus/rhinovirus influenza A and B, human metapneumovirus, parainfluenza and RSV.

Statistical analyses were performed at the patient and specimen level using Stata/SE version 10.0 (StataCorp LP, College Station, TX, USA). Clinical and demographic information provided on PHOL’s laboratory requisition was used for this investigation; patient charts were not available for review as PHOL is the provincial reference laboratory and as such has no direct access to patient records. Specimen collection date was used for these analyses and in the event that this was missing, it was replaced by the date when the specimen was received at PHOL. Data were transformed from specimen to patient level by probabilistic patient linkage; when discrepant results (both a positive and a negative result) were identified for the same patient, the positive result took priority over the negative one. In the event more than one specimen was submitted per patient, specimen collection date of the first specimen submitted was used and the setting that indicated the highest clinical severity (e.g. hospitalized vs. community dwelling) was used to describe disease severity. Percent positivity and descriptions of EV-D68 cases by timing of specimen collection, age, sex, setting, symptoms and local health unit of residence were analysed at the patient level. Reports of other viruses identified in addition to EV-D68 and specimen type were described at the specimen level. Bivariate and multivariate logistic regression analyses were performed at the patient level to compare cases and controls in terms of age, sex, settings, symptoms and timing of specimen collection. P-values and odds ratios with a 95% confidence interval (CI) were reported.

## Results

### Patient level analyses

**Fig 1:**
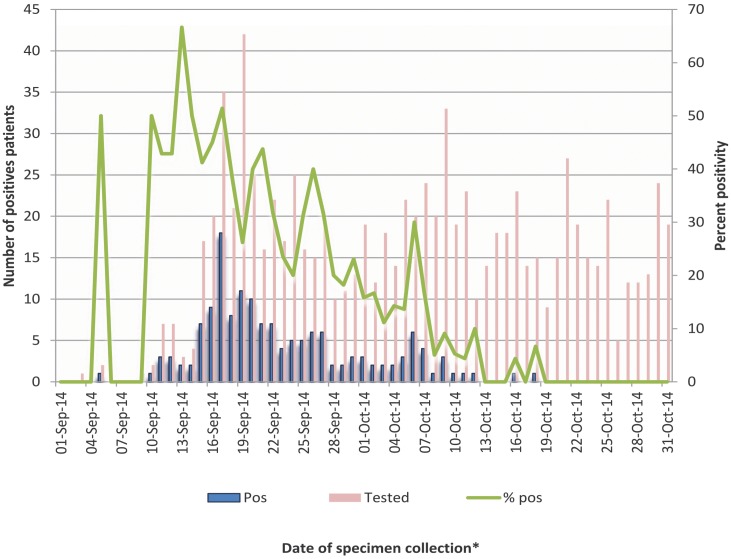
Number of positive patients and percent positivity for EV-D68, PHOL/NML testing, September 1 to October 31, 2014. *Date when specimen was collected was used for this analysis and date when specimen was received in laboratory was used in the event the date of specimen collection was missing.

There were a total of 907 patients tested for EV-D68 at PHOL from September to October 2014; of these patients 153 (16.9%) tested positive for the virus. The date for which the first EV-D68 positive specimen from a case was collected was September 4th, 2014. September 16^th^, 2014 was the specimen collection date for the greatest number of cases and highest test positivity, with 18/35 (51.4%) cases (Fig 1).

After September 16^th^, the number of EV-D68 cases begins to decline; the specimen collection date for the last case was October 17^th^, 2014. EV-D68 was not identified in any of the patients tested before September (n = 18 patients) or after October (n = 168 patients); therefore, only data from September and October were included in the final analyses. In bivariate analysis, compared to controls, EV-D68 cases were more likely to occur in September than October (OR 9.47; 95% CI 5.99 to14.36) (Table 1).

**Table 1:** Crude and adjusted odds ratio of patient characteristics by EV-D68 status using test negatives as the reference.

Variables category		EV-D68	Negative	Crude OR(95% CI)n = 907	Adjusted OR(95% CI)n = 904 ^α^
		Counts (%)n = 153	Counts (%)n = 754		
**Age group (in years)**	20+	7 (4.6)	122 (16.2)	1.00	1.00
	0–4	75 (49)	447 (59.3)	2.92 (1.31–6.50)*	2.61(1.02–6.70)*
	5–9	50 (32.6)	114 (15.2)	7.64 (3.32–17.55)*	5.67 (2.14–15.05)*
	10–19	21 (13.7)	71 (9.4)	5.15 (2.08–12.73)*	4.72 (1.65–13.48)*
**Sex**	Female	54 (35.3)	325 (43.3)	1.00	1.00
	Male	99 (64.7)	426 (56.7)	1.39 (0.97–2.01)	1.25 (0.84–1.87)
**Setting**	Physician's office	12 (7.8)	128 (17.0)	1.00	1.00
	Emergency department	2 (1.3)	12 (1.6)	1.77 (0.35–8.89)	0.72 (0.13–4.05)
	Hospital	136 (88.9)	574 (76.2)	2.52 (1.35–4.70)*	1.41 (0.71–2.79)
	ICU	2 (1.3)	10 (1.3)	2.13 (0.41–10.88)	5.99 (0.89–39.98)
	Other ^β^	1 (0.7)	30 (3.9)	0.35 (0.44–2.84)	0.5 (0.65–4.73)
**Month**	October	28 (18.3)	509 (67.5)	1.00	1.00
	September	125 (81.7)	245 (32.5)	9.27 (5.99–14.36)*	8.07 (5.15–12.64)*

^α^ Three patients were missing sex information and therefore they were removed from the crude sex analysis and adjusted logistic regression analysis.

^β^ Other settings category includes those from institutions and those with no setting reported.

* Indicates significant OR.

The median age of patients (i.e. cases and controls) tested was 3 years (range 3 weeks to 94 years). More than half of patients tested (686/907; 75.6%) were less than 10 years old (Table 1). In bivariate analysis, using patients 20 years of age and over as the reference, cases were more likely than controls to be less than 20 years of age; the effect peaked at ages 5–9, (OR 7.58; 95%CI 3.30 to 17.40). Of those patients for whom sex was reported, 525 (58.0%) were male; sex was not reported for three patients. Cases and controls did not differ by sex (p>0.05).

In patients for whom a setting type was reported, most patients (710/880; 80.6%) were hospitalized and (12/880; 1.4%) were admitted to an intensive care unit (ICU). The remainder of patients were either seen in a community physician’s office (140/880;15.9%), in the emergency department (14/880; 1.6%) or were in an institution (4/880;0.2%). In bivariate analysis when compared to controls, cases were more likely to be hospitalized as opposed to seeking care at a physician’s office (OR 2.51; 95%CI 1.35 to 4.68).

Among all patients tested for EV-D68, 609/907 (67.1%) reported at least one symptom; 463 (76%) reported undefined respiratory symptoms (i.e. when the “respiratory symptom” option was selected on the laboratory requisition without providing further clinical details), 277 (45.5%) reported fever or chills, 51(8.3%) reported cough, 37 (6.1%) reported shortness of breath, 9 (1.5%) reported headache and six (0.9%) reported vomiting. In addition, 20 (3.3%) were reported to have pneumonia and 50 (8.2%) reported as having asthma. Comparing cases to controls in regards to symptoms, cases were more likely to report at least one symptom (75.8% versus 67.1%, respectively; (p-value <0.05)). In comparison to controls, undefined respiratory symptoms were reported more frequently in cases than controls, (81% versus 60.5%, respectively, (p-value <0.001)).

In the logistic regression model adjusting for age, sex, setting and time from symptom onset to specimen collection, individuals younger than 20 years of age were more likely to be diagnosed with EV-D68 compared to those 20 and over (Table 1); this effect peaked at ages 5–9 (OR 5.67; 95% CI 2.14 to 15.05). In addition, cases were more likely to be identified in September than October (OR 8.07; 95% CI 5.15 to 12.64).

**Fig 2:**
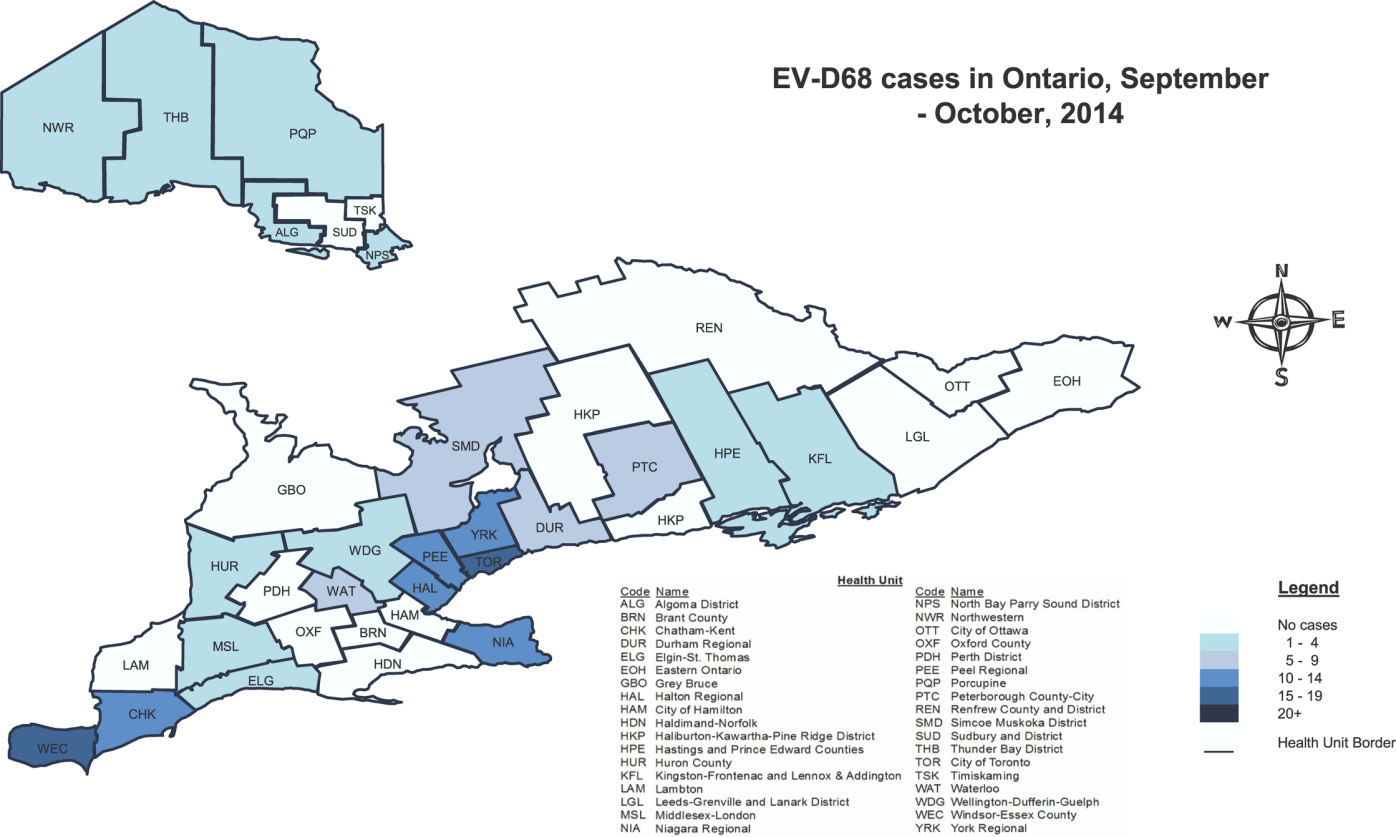
Geographical distribution of EV-D68 cases in Ontario, PHOL/NML, September 1 to October 31, 2014. A) Health unit reflects the jurisdiction in which the patient resides. In the event this was not available, health unit of the specimen submitter was used.

The local health unit of residence where the greatest numbers of patients tested for EV-D68 resided were Peel Regional with 124/907 (13.6%) patients, City of Toronto 121/907 (13.3%), Windsor Essex County (WEC) 80/907(8.9%) and Halton Regional 63/907 (7%). The remaining 32 health units each represented less than 5% of the other patients tested. The greatest number of cases resided in south western Ontario; the city of Toronto was the health unit of residence for 26/153 (17%) of cases, WEC for 20/153 (13.1%), York Regional 13/153 (8.5%), Peel 13 (8.5%), Halton Regional 13/153 (8.5%), Niagara Regional 11/153 (7.2%) and Chatham-Kent (CHK) for 10/153 (6.5%) cases (Fig 2).

In proportion to each health unit’s population in Ontario, the highest incidence rate was observed in CHK with 9.1 cases per 100, 000 population followed by WEC with 5 cases per 100,000 population. However, when comparing the proportion of patients tested by local health units proportionate to Ontario’s population, both health units tested more specimens relative to their proportion of Ontario’s population (p-value<0.001).

### Specimen level analysis

Of the 959 respiratory specimens tested, EV-D68 was identified in 155. Thirty-eight patients had more than one specimen submitted; two patients had two positive specimens identified. Of all specimens tested for EV-D68, 408 (42.5%) were tested by NML only, 489 (50.9%) were tested by PHOL, and 62 (6.4%) were tested by both laboratories. Percent positivity by laboratory was 144/470 (30.6%) for NML and 18/551 (3.2%) for PHOL. It should be noted that NML tested most of the specimens 370/470 (78.7%) in September during peak activity, while PHOL tested most of specimens 490/551 (88.9%) in October, once peak activity had declined.

Specimen source for EV-D68 positive cases was either nasopharyngeal swab, respiratory aspirate, throat swab or nasal swab. Percent positivity by specimen source was 146/869 (16.8%) for nasopharyngeal swab, 5/10 (50%) for respiratory aspirate, 3/17 (17.7%) for throat swab, and 1/20 (5%) for nasal swab. EV-D68 was not detected in any of the five cerebrospinal fluid (CSF) specimens submitted for testing. Similarly, EV-D68 was not detected in any specimens collected from bronchoalveolar lavage (BAL) (n = 9), sputum (n = 3), or stool (n = 9). The other specimen types submitted for EV-D68 testing included poorly defined specimen types e.g. fluid or swab or aspirate (n = 27).

Of all 155 EV-D68 positive specimens, 154 were also tested by viral culture and / MRVP as per routine laboratory testing at PHOL. Of the 148 specimens tested by viral culture, 4 (2.7%) specimens were classified as “entero-like virus”, 2(1.4%) specimens as “enterovirus, probably rhinovirus” and 1(0.7%) specimen as parainfluenza 2 virus. The remaining 141(95.3%) specimens were negative for any respiratory virus. Of 89 EV-D68 positive specimens tested by MRVP, 29 (32.6%) specimens were classified as rhinovirus, 1(1.1%) specimen as rhino and enterovirus, 3(3.4%) specimens as rhino and parainfluenza 4 virus, 1 (1.1%) specimen as parainfluenza and 1(1.1%) specimen as adenovirus. More than half 54 (60.7%) of EV-D68 specimens tested by MRVP were negative for any virus. In total, co-infection of EV-D68 with adenovirus or parainfluenza viruses was detected in 6 specimens.

## Discussion

EV-D68 virus activity was identified from September to October, 2014 in Ontario, Canada. EV-D68 was detected in 17% of patients on whom testing was performed, with peak activity occurring in September. Testing of specimens submitted prior to September 2014 and after October 2014 did not identify EV-D68. From 1987 until 2014, EV-D68 had been rarely reported in the US [20], however the number of cases reported in 2014 increased sharply, and was much higher than previously reported [21]. In Ontario, EV-D68 may have circulated prior to 2014 not all enteroviruses are typed and not all test methods are able to distinguish between rhinovirus and enterovirus. Routine viral culture and MRVP testing did not detect enterovirus and EV-D68 viruses in 95.9% and 60.3% of PCR-positive specimens, respectively. In addition neither viral culture nor MRVP could distinguish EV-D68 from rhinovirus in 1.3% and 36.4% of specimens tested by each method, respectively. Hence it is likely that EV- D68 has been underdiagnosed when only viral culture or MRVP test methods were used [22].

Consistent with previous reports, in Ontario EV-D68 was most commonly found in younger children as more than 50% of cases were identified in children less than 5 years of age (7,9–14). When adjusted for age, sex, setting and date of specimen collection, the likelihood of having EV-D68 was higher for individuals less than 20 years of age compared to over 20, peaking for the 5–9 year old age group. While this may be indicative of the population in whom EV-D68 is most frequently detected, but may also represent a testing bias as three-quarters of patients tested for EV-D68 were less than 10 years of age. Consistent with what has been reported elsewhere, males were more likely to be cases than controls [2], however this did not achieve statistical significance.

Higher detection of EV-D68 in hospitalized patients was reported by other studies [23]. In our investigation, most patients tested for EV-D68 were hospitalized and this may reflect the fact that hospitalized patients are more likely to be tested than patients seeking community based care. However, in multivariate analysis when adjusted for age, sex, setting and date of specimen collection, cases were not more likely to be hospitalized than controls.

Symptoms reported among patients tested for EV-D68 included fever, chills, cough and shortness of breath. When we compared cases and controls in regards to reported symptoms, undefined respiratory symptoms were more likely to be reported for EV-D68 cases as opposed to controls. Neurological symptoms such as muscle weakness or acute flaccid paralysis were not reported. A wide range of disease severity has been reported in the literature, varying from mild to more severe illness particularly among children [7,9]. Based on the limited information available to PHOL through the laboratory requisition, we could not determine disease severity for EV-D68 cases. In addition, neither cases nor controls were followed up to determine disease trajectory, therefore we could not determine if clinical patient’s condition deteriorated after specimen collection.

All EV-D68 viruses were detected in respiratory specimens including nasopharyngeal, respiratory aspirate, throat swabs and nasal swabs; however this should be interpreted with caution as the number of non-respiratory specimens submitted for testing was small (n = 14 patients). Infection with EV-D68 predominantly resulted in respiratory symptoms. EV-D68 was not detected in stool, CSF, sputum or BAL. This is congruent with what has previously reported elsewhere [7]; notably EV-D68 has been seldom reported in CSF [13,24], or stool [25]. We also found cases who were co-infected with EV-D68 and parainfluenza or adenovirus, which has not been previously described in other studies.

There are some limitations to this investigation. Firstly, PHOL was not the only laboratory that performed EV-D68 testing in Ontario since the Regional Virology Laboratory in Hamilton Ontario and the Children’s Hospital of Eastern Ontario in Ottawa also performed EV-D68 testing. As well, some hospitals may have forwarded their specimens directly to NML. Hence, these results do not represent all EV-D68 detections in Ontario. In addition, physicians may not routinely test patients presenting with respiratory symptoms and some patients with EV-D68 with mild symptoms likely did not present for care; therefore cases reported in this investigation underestimate the true incidence of this virus.

Secondly, for the purposes of this investigation, testing for EV-D68 was performed by PHOL and NML. Different test methods for enterovirus detection were used by each laboratory, which may have different sensitivity and specificity and consequently may have affected the number of positive results reported in this study. Thirdly, information about at-risk groups (e.g. children, particularly those with asthma) requiring hospitalization as a result of EV-D68 had been communicated broadly to clinicians. Hence, it is not possible to determine if requests for testing for EV-D68 by health care providers was requested more frequently for these specific population groups as opposed to broadly testing individuals of any age regardless of setting type. In addition, some clinicians may have been more likely to test than others, which would impact the number of EV-D68 positive specimens attributed to any given health unit. Finally, reviewing patients’ charts would have been the optimal method by which to obtain clinical information such as patients’ symptoms and clinical outcomes. At PHOL, we are reliant on information supplied by clinicians on the laboratory requisition. Unfortunately, symptoms and patient’s setting were not consistently reported on the requisition. In addition, even when reported, they reflect one specific period during the clinical course of disease. As a result, we are unable to report on clinical severity of disease, and/ or clinical outcome.

## In Conclusion

Presence of EV-D68 was identified among young children during September-October 2014 in Ontario, with positive cases occurring more frequently in September. Although most of the patients were hospitalized there was no difference in hospitalization status between cases and controls. Most cases reported respiratory symptoms, and the virus was only detected in respiratory specimens. In order to better understand the epidemiology of this virus, surveillance for EV-D68 should include testing of symptomatic individuals regardless of treatment setting (including ambulatory care settings) patient age, along with collection and analysis of comprehensive clinical and epidemiological data.

## Supporting Information

S1 Dataset Ontario patients who tested for Enterovirus D68, PHOL, 2014. Ontario patients who tested for EVD-68 at PHOL are included in this dataset. To protect the patient’s privacy, the data were aggregated and de-identified from all personal identifiers including (health card number, birth date, health unit and address). A generic patient id was created to identify unique patients.
(CSV)
